# Li_7_La_3_Zr_2_O_12_ Nanofiber-Modified Polypropylene Composite Separator for High-Performance Lithium-Ion Batteries

**DOI:** 10.3390/ma19183991

**Published:** 2026-09-19

**Authors:** Yuxing Liu, Lan Xu

**Affiliations:** 1 National Engineering Laboratory for Modern Silk, College of Textile and Engineering, Soochow University, Suzhou 215123, China; 2 Jiangsu Engineering Research Center of Textile Dyeing and Printing for Energy Conservation, Discharge Reduction and Cleaner Production, Soochow University, Suzhou 215123, China; 3 Jiangsu Advanced Textile Engineering Technology Center, Nantong 226007, China

**Keywords:** lithium-ion batteries, composite separators, electrospinning, nanofibers, garnet-type LLZO

## Abstract

Separators dominate lithium-ion battery (LIB) safety by isolating electrodes, guiding Li^+^ transport and avoiding short circuits. However, commercial polypropylene (PP) separators suffer from inferior electrolyte wettability, severe thermal shrinkage, and insufficient suppression of lithium dendrite growth. Herein, garnet-type Li_7_La_3_Zr_2_O_12_ (LLZO) nanofibers were synthesized via spherical section free surface electrospinning (SSFSE) followed by calcination. LLZO nanofiber-modified PP separators (LLZO@PP) were fabricated by coating a single side of PP separators with the as-synthesized LLZO nanofibers. Systematic morphological, structural and electrochemical characterizations reveal that the modified LLZO@PP separators exhibit improved thermal stability and electrolyte wettability compared with the pristine PP separator. The optimized LLZO@PP separator delivers a high ionic conductivity of 0.571 mS cm^−1^ and a wide electrochemical window of 4.8 V at 25 °C. Furthermore, the Li//LiFePO_4_ half-cell assembled with the optimal LLZO@PP separator achieves a capacity retention of 90.21% after 100 cycles at 0.5 C. This work offers a facile and scalable route to construct LLZO nanofiber-modified composite separators, which can effectively improve the safety and superior electrochemical performance of high-performance LIBs.

## 1. Introduction

With the rapid development of electric vehicles, wearable electronics, and large-scale renewable energy power stations, lithium-ion batteries (LIBs) have dominated modern electrochemical energy storage systems due to their superior energy density, stable cycling performance, and mature industrial manufacturing technologies [1,2,3]. Nevertheless, frequent incidents of battery thermal runaway, internal short circuits, and lithium dendrite penetration severely limit the high-safety and long-cycle serviceability of commercial LIBs under high-rate and high-temperature operating conditions [4]. Battery safety is collectively determined by cathode and anode materials, electrolyte components, and separators. As a core battery component, separators serve to isolate the positive and negative electrodes, regulate lithium-ion migration behaviors, and inhibit internal short-circuit reactions. Alongside cathodes, anodes and electrolytes, separators are essential determinants of the intrinsic safety and electrochemical compatibility of LIBs [5,6,7,8].

Currently, polypropylene (PP) separators dominate the commercial polyolefin separators for LIBs, featuring low production costs and a well-established dry-stretching manufacturing technology. Despite these merits, dry-processed PP separators exhibit highly anisotropic mechanical properties: high tensile strength along the machine direction yet unsatisfactory puncture resistance and inferior mechanical performance in the transverse direction. Apart from mechanical drawbacks, commercial PP separators suffer from inherent shortcomings including weak electrolyte affinity, low electrolyte uptake, and high lithium-ion migration resistance, which significantly impair their practical service performance [9,10]. Furthermore, PP separators suffer from severe thermal shrinkage at elevated temperatures, which easily causes electrode contact and subsequent thermal runaway. Exacerbating these deficiencies, the chemically inert surface of PP separators cannot effectively inhibit the nucleation and growth of lithium dendrites during long-term cycling, thereby increasing the risk of battery failure. Accordingly, surface modifications have become the most feasible and cost-effective strategy to improve the comprehensive performance of commercial PP separators while fully compatible with existing industrial production lines [11].

Garnet-type Li_7_La_3_Zr_2_O_12_ (LLZO) has recently emerged as a high-efficiency modifier for LIB separators due to its high intrinsic lithium-ion conductivity, wide electrochemical stability window, and extremely low thermal expansion coefficient [12,13,14]. Numerous studies have demonstrated that LLZO modification can effectively mitigate the inherent defects of polyolefin separators. Most existing studies adopt LLZO nanoparticle coating to functionalize PP separators, which improves the thermal stability and electrolyte wettability of composite separators to a certain extent. However, discrete LLZO nanoparticles tend to agglomerate on separator surfaces, forming discontinuous lithium-ion transport pathways and uneven coating layers. This phenomenon limits further improvements in ionic conductivity and lithium dendrite suppression ability of modified separators [15,16,17,18]. Furthermore, particle-assembled coating layers lack sufficient structural continuity, resulting in limited mechanical reinforcement and inferior long-cycle interfacial stability.

Compared with conventional discrete LLZO powders, high-aspect-ratio LLZO nanofibers can construct continuous and interconnected three-dimensional networks on the separator surface, effectively alleviating the structural defects of nanoparticle-modified separators [19]. Such continuous networks create unobstructed fast Li^+^ transport pathways to improve ionic conductivity and the Li^+^ transference number, while providing abundant polar active sites to enhance electrolyte retention and lower interfacial impedance [20]. Notably, cubic garnet-type LLZO is intrinsically unable to fully block lithium penetration. Under operating conditions, lithium metal tends to propagate through polycrystalline or single-crystal LLZO along grain boundaries, microcracks, and structural defects [21]. In this liquid-electrolyte battery system, surface-decorated LLZO nanofibers primarily homogenize the interfacial Li^+^ flux and improve thermal dimensional stability, rather than serving as bulk solid electrolytes for full dendrite suppression.

Despite the remarkable advantages of LLZO nanofiber modification, current research remains limited by insufficient optimization of electrospinning and sintering parameters. Furthermore, the structure–performance discrepancies between nanofiber- and particle-coated PP separators have not been thoroughly elucidated. In view of the above research gaps, this work successfully fabricated uniform cubic-phase LLZO nanofibers via spherical section free surface electrospinning (SSFSE) combined with high-temperature calcination [22]. The PVP concentration and sintering temperature were systematically optimized to enable the synthesis of high-quality nanofibers. Thereafter, a facile blade-coating method was employed to construct homogeneous LLZO nanofiber network coatings on commercial PP separators. The microstructure, mechanical robustness, thermal stability, and electrochemical performances of the optimized LLZO nanofiber-modified PP separator (LLZO@PP) were comprehensively investigated. The structural and performance superiority of nanofiber coating over conventional nanoparticle coating was well demonstrated. Benefiting from the continuous nanofiber network, LLZO@PP exhibits improved electrolyte wettability, ionic conductivity and thermal dimensional stability. This work provides a feasible laboratory-scale route for the rational design and performance optimization of advanced composite separators for high-performance LIBs.

## 2. Preparation of LLZO@PP Separators

**Figure 1:**
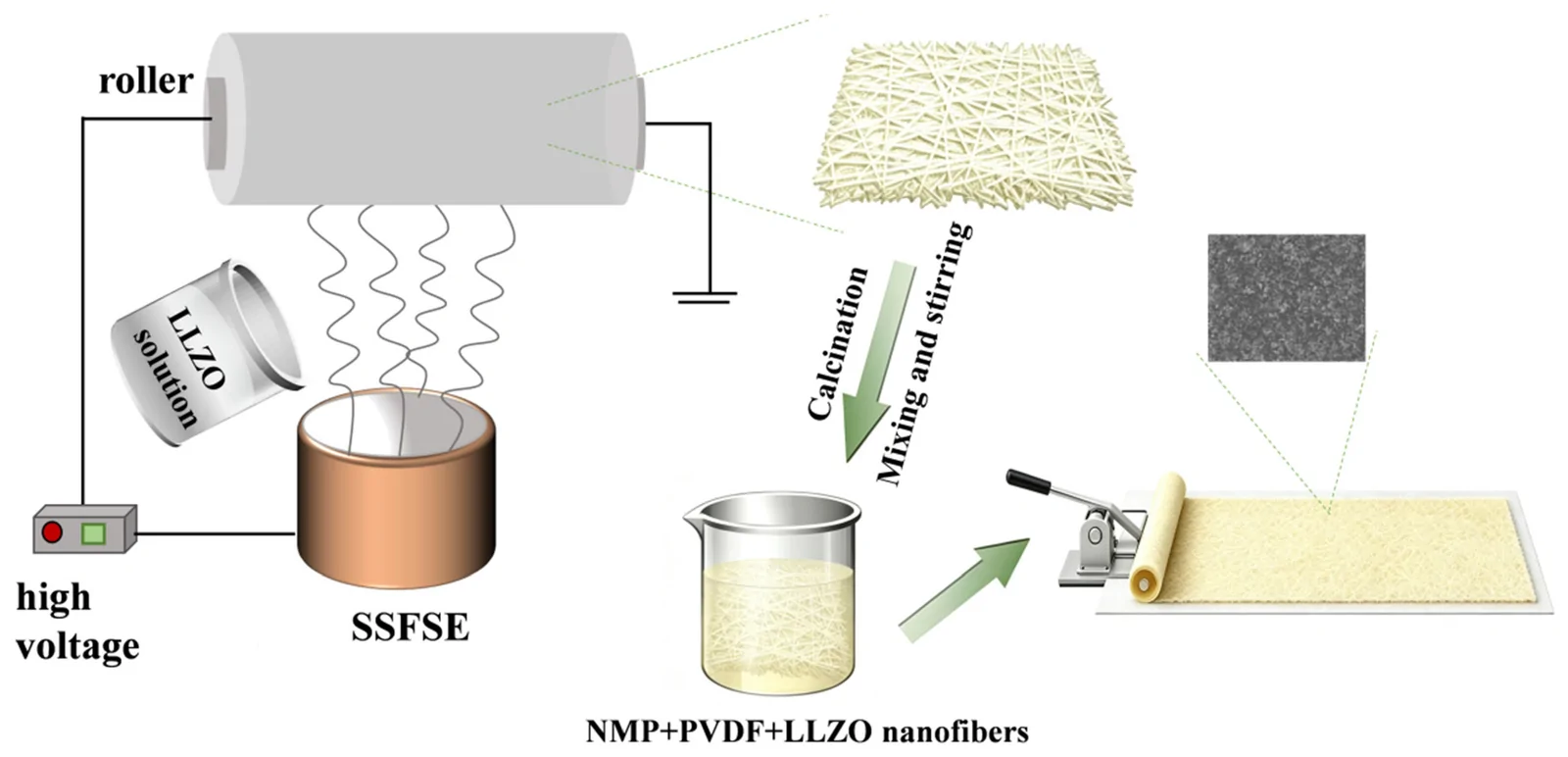
Fabrication procedures of LLZO@PP separators.

As illustrated in Figure 1, LLZO precursor fibers were fabricated by the SSFSE technique using spinning solutions with varying PVP concentrations (8 wt%, 10 wt%, and 12 wt%). The SSFSE conditions were set to a spinning voltage of 60 kV, a collector distance of 220 mm, and a drum rotation speed of 600 r/min. All SSFSE experiments were carried out at temperatures of 20–30 °C and a relative humidity of 30–40%. The as-spun precursor fibers were first pre-oxidized at 280 °C for 2 h, followed by high-temperature sintering in air at 650–800 °C for another 2 h with a heating rate of 2 °C min^−1^, ultimately yielding LLZO nanofibers.

Subsequently, 0.164 g of polyvinylidene fluoride (PVDF) powder was dissolved in 2 g of N-methyl-2-pyrrolidone (NMP) and ultrasonically dispersed at room temperature for 0.5 h. As-prepared LLZO nanofibers or commercial LLZO nanoparticles (particle size: 300 nm, purity: 99.9%, denoted as C-LLZO) were incorporated at a solid content of 20 wt%, followed by 12 h room-temperature stirring to form homogeneous slurries. The well-blended slurries were blade-coated onto commercial PP separators. After vacuum drying at 30 °C for 24 h, the target composite separators were obtained, named LLZO@PP and C-LLZO@PP, respectively. Detailed information regarding raw materials, spinning solution preparation, material characterization, battery assembly, and electrochemical measurement procedures is provided in the Supplementary Materials.

## 3. Results and Discussion

### 3.1. Effects of PVP Concentration

**Figure 2:**
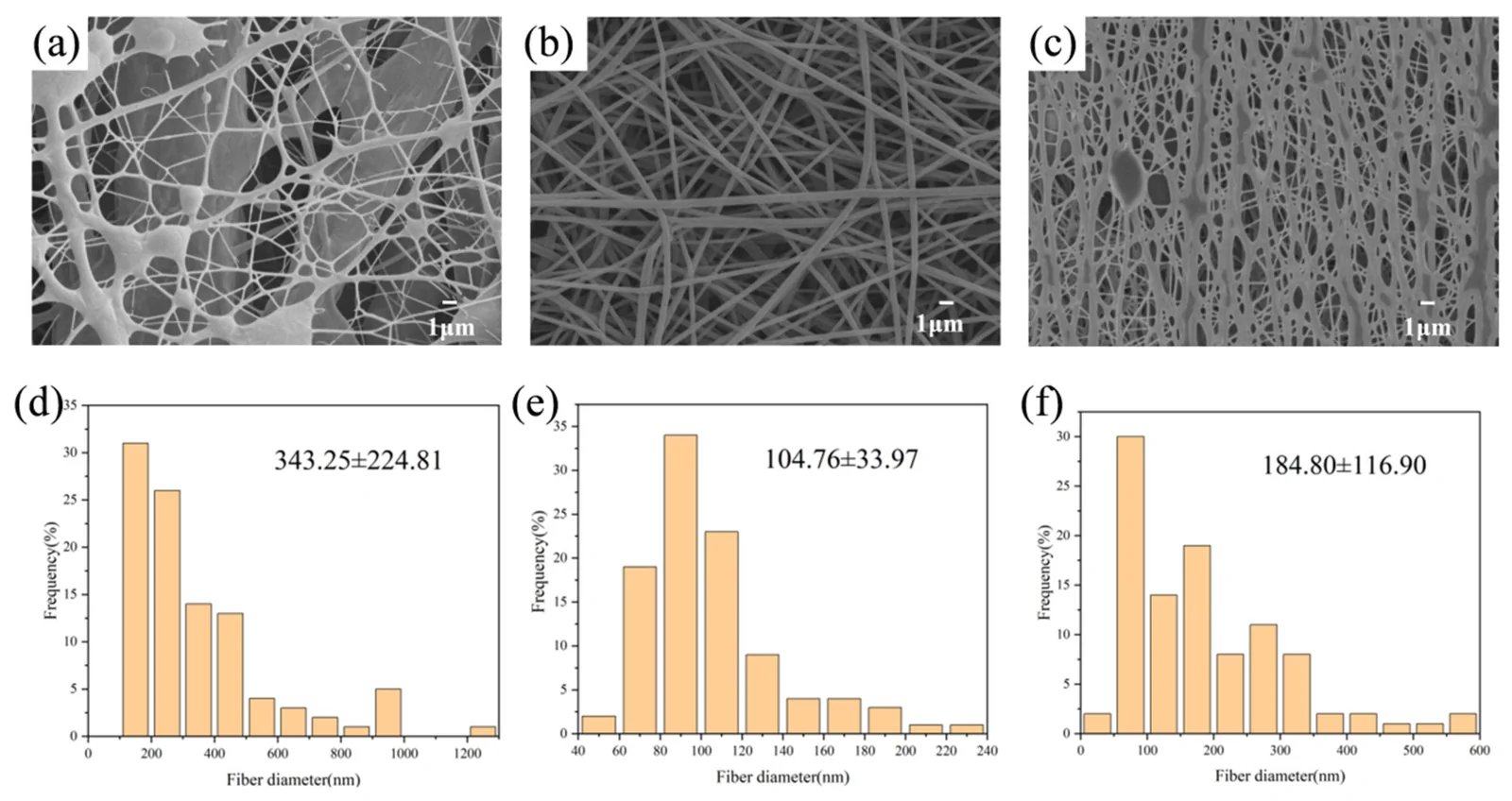
Scanning electron microscopy (SEM) images of LLZO precursor fibers obtained at different PVP concentrations: (**a**) 8 wt%, (**b**) 10 wt%, (**c**) 12 wt%; (**d**–**f**) corresponding fiber diameter distributions.

Figure 2a shows abundant droplets and bead-on-string structures within electrospun samples prepared using a PVP concentration of 8 wt%. This phenomenon originates from the low solution viscosity caused by insufficient polymer content. The electrostatic force far exceeds the solution surface tension, inducing unstable jet ejection and incomplete solvent volatilization. The statistical diameter distribution (Figure 2d) demonstrates that the fibers obtained at 8 wt% PVP exhibit an average diameter of 343.3 ± 224.81 nm, accompanied by extremely broad diameter dispersion. As the PVP concentration rises, droplet formation is gradually suppressed. At 10 wt% PVP, uniform, droplet-free, and continuous LLZO nanofibers are obtained, with distinct boundaries between adjacent fibers (Figure 2b). The corresponding diameter histogram (Figure 2e) confirms the narrowest size distribution, with a minimum average fiber diameter of 104.76 ± 33.97 nm. Further raising the PVP concentration to 12 wt% significantly increases fiber diameter and causes substantial inter-fiber adhesion (Figure 2c). Its diameter distribution (Figure 2f) exhibits an average fiber diameter of 184.8 ± 116.9 nm with broader size variation. This trend is mainly attributed to the excessive solution viscosity at high PVP loading, which constrains jet stretching under the electric field. Residual solvent also leads to fiber adhesion before complete drying, further enlarging fiber diameter and degrading fiber uniformity. Accordingly, 10 wt% PVP concentration is selected as the optimal concentration for preparing high-quality LLZO nanofibers.

### 3.2. Effect of Sintering Temperature

**Figure 3:**
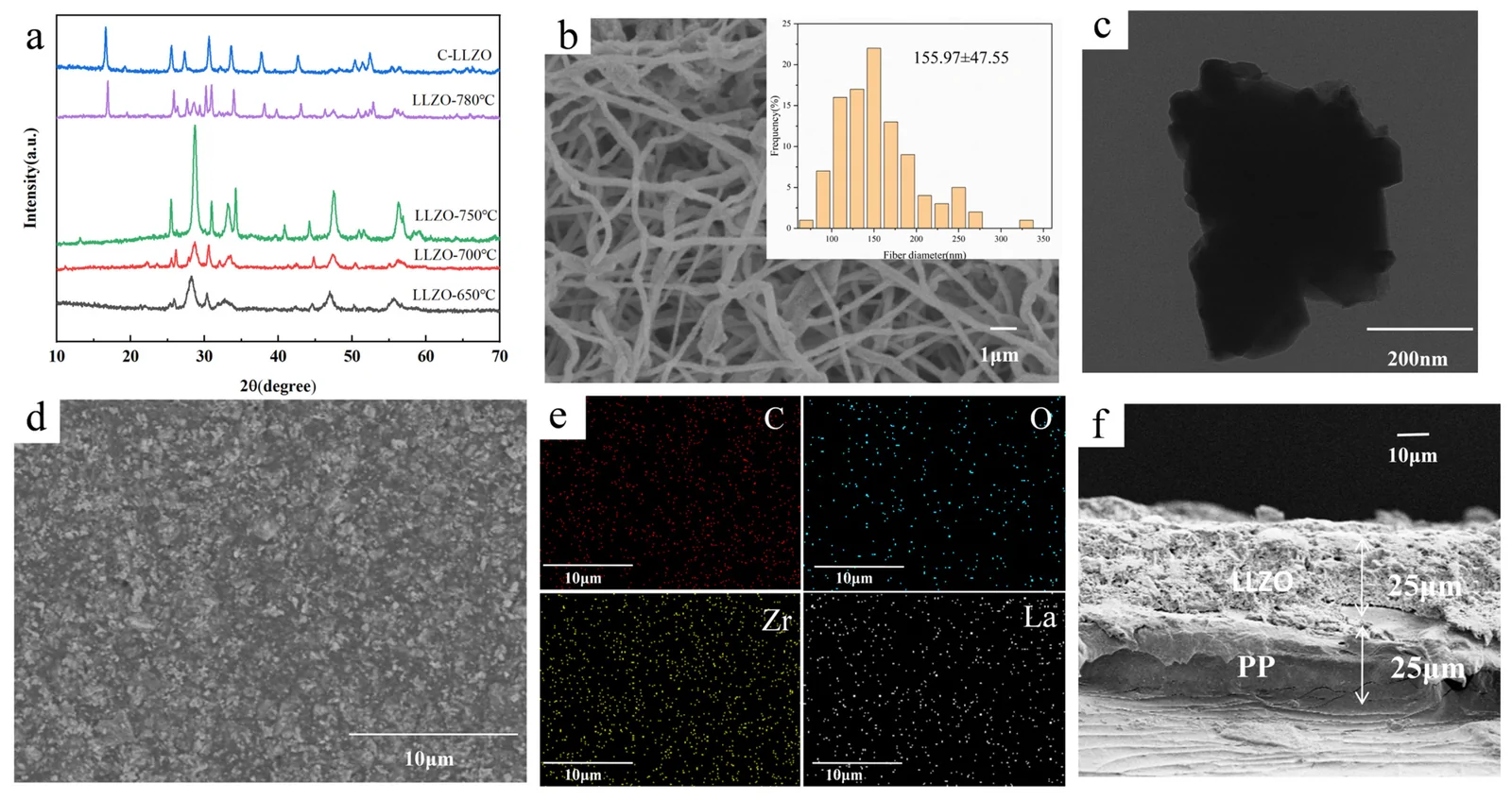
(**a**) XRD patterns of C-LLZO and LLZO nanofibers sintered at different temperatures. (**b**) SEM image and corresponding fiber diameter distribution, and (**c**) TEM image of LLZO nanofibers sintered at 780 °C. (**d**) Top-view SEM image, (**e**) EDS elemental mapping, and (**f**) cross-sectional SEM image of LLZO@PP.

X-ray diffraction (XRD) characterization was performed to analyze the phase structure and crystallinity of LLZO nanofibers sintered at different temperatures (Figure 3a). The samples sintered at 650 °C and 700 °C display weak and broad diffraction peaks. Despite the partial overlap of several diffraction peaks with those of commercial cubic-phase C-LLZO, such weak and broad characteristic reflections indicate low crystallinity of the samples. At these temperatures, tetragonal-phase LLZO remains the dominant phase, and a well-defined cubic-phase lattice structure failed to form. When the sintering temperature rises to 750 °C, both the number and intensity of diffraction peaks increase noticeably with narrowed peak width, demonstrating significant improvement in crystallinity. Upon further increasing the sintering temperature to 780 °C, the position and distribution of diffraction peaks match well with the standard pattern of cubic LLZO (JCPDF 80-0457), confirming the successful formation of the cubic phase [23,24]. These results verify that stable cubic-phase LLZO nanofibers can be effectively fabricated via sintering at 780 °C.

### 3.3. Morphological and Structural Characterization

As illustrated in Figure 3b–f, comprehensive microscopic characterizations were performed to investigate the microstructures of LLZO nanofibers and LLZO@PP. After sintering at 780 °C, the LLZO nanofibers retain intact continuous structures without obvious fractures and exhibit a uniform diameter distribution, thereby constructing a well-interconnected porous framework (Figure 3b). TEM observation (Figure 3c) further displays the fine microstructure of individual LLZO nanofibers, with an average fiber diameter of approximately 250 nm, which supports subsequent analysis of LLZO grain size. The top-view SEM image of LLZO@PP (Figure 3d) suggests that LLZO nanofibers are fully and evenly covered on the PP substrate surface, without severe localized particle agglomeration or exposed substrate regions, demonstrating outstanding homogeneity of the modified layer. The corresponding EDS elemental mapping (Figure 3e) verifies the uniform distribution of C, O, Zr, and La elements across the coating region. Among them, Zr and La serve as the characteristic constituent elements of garnet-type LLZO, while the carbon signal mainly derives from the underlying PP substrate. These observations confirm the uniform immobilization of LLZO nanofibers on the separator surface. The cross-sectional SEM image (Figure 3f) intuitively verifies robust interfacial adhesion between the LLZO nanofibers and PP substrate, forming a uniform LLZO coating with a thickness of approximately 25 μm, which is comparable to the thickness of the PP substrate. Overall, these microscopic results adequately validate the successful fabrication of homogeneous LLZO-modified PP separators via a facile blade-coating strategy.

**Figure 4:**
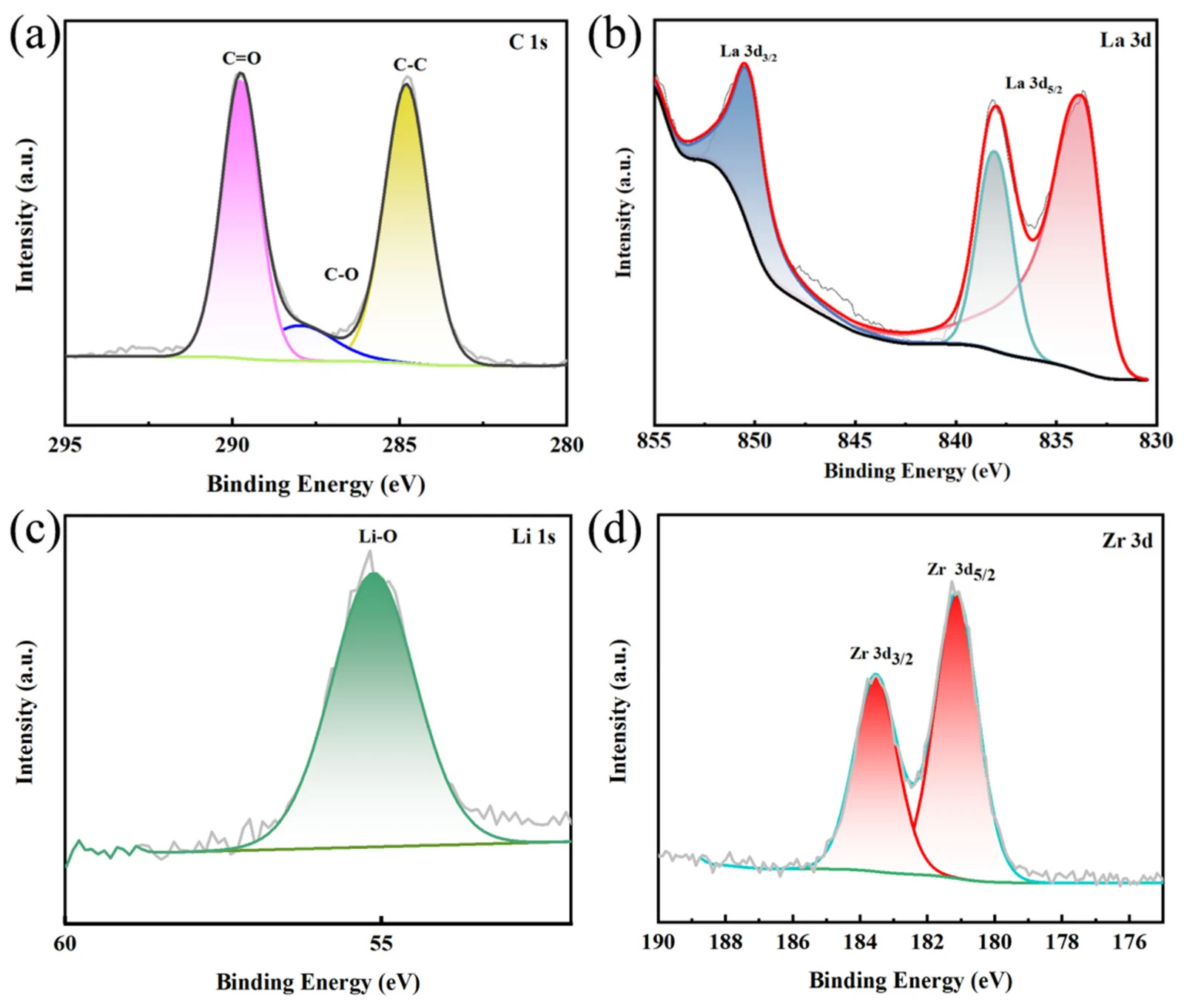
(**a**) C 1s, (**b**) La 3d, (**c**) Li 1s, and (**d**) Zr 3d XPS spectra of LLZO nanofibers.

X-ray photoelectron spectroscopy (XPS) was employed to analyze the surface elemental composition and chemical bonding states of the synthesized LLZO nanofibers (Figure 4). The survey spectrum (Figure S1) confirms the existence of C, La, Li, and Zr. The high-resolution C 1s spectrum (Figure 4a) can be deconvoluted into three distinct peaks assigned to C-C (284.8 eV), C-O (286.2 eV), and C=O (288.7 eV) bonds. This observation demonstrates that LLZO nanofibers may spontaneously react with moisture and carbon dioxide in ambient air, forming a thin carbonate passivation layer on the sample surface. As displayed in the high-resolution La 3d spectrum (Figure 4b), two characteristic spin-orbit doublets of La 3d_3/2_ and La 3d_5/2_ are clearly resolved, accompanied by prominent satellite shake-up peaks between the two main peaks. The binding energies of these La 3d peaks match the standard trivalent La state in garnet-type LLZO, confirming the La^3+^ chemical environment [25]. For the Li 1s spectrum (Figure 4c), the dominant peak centered near 55 eV is attributed to Li-O bonding within the LLZO crystal lattice, directly proving Li-O coordination within the garnet framework. Regarding the Zr 3d spectrum (Figure 4d), two well-resolved spin-orbit-split peaks for Zr 3d_3/2_ and Zr 3d_5/2_ are clearly observed, whose binding energies correspond to the typical tetravalent Zr state in stoichiometric garnet LLZO [26]. Overall, the XPS characterization systematically verifies the complete elemental composition and intrinsic chemical bonding configuration of the as-prepared LLZO nanofibers, while revealing unavoidable surface carbonation when LLZO is exposed to ambient air.

### 3.4. Physical Properties of Separators

**Figure 5:**
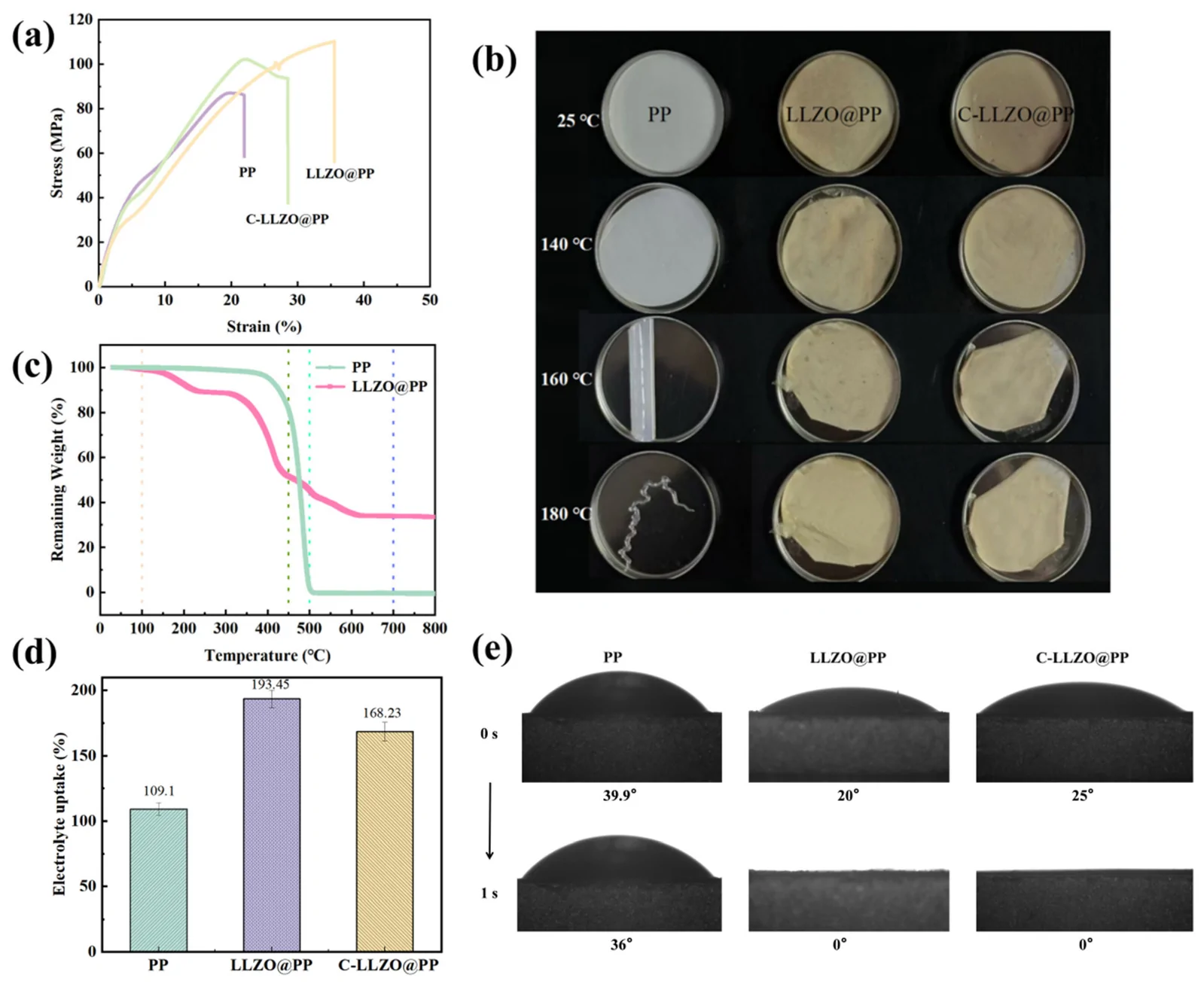
(**a**) Mechanical properties, (**b**) thermal shrinkage behavior, (**c**) TG curves, (**d**) electrolyte uptake, and (**e**) electrolyte contact angles of different separators.

All samples were stretched along the intrinsic stretching direction of the PP separator to eliminate mechanical deviations induced by varied testing orientations, and all measurements were performed in triplicate (n = 3). Figure 5a presents the representative stress–strain curves of different separators. The tensile strength and elongation at break of the three separators were calculated using Equations (S1) and (S2). The pristine PP separator exhibits a tensile strength of 57.75 ± 2.14 MPa and an elongation at break of 21.96 ± 1.21%. After LLZO modification, C-LLZO@PP presents improved mechanical performance, with a tensile strength of 91.82 ± 3.07 MPa and an elongation at break of 28.53 ± 1.45%. Notably, LLZO@PP delivers further enhanced mechanical performance, achieving a tensile strength of 109.42 ± 3.68 MPa and an elongation at break of 35.55 ± 1.73%, both of which are substantially higher than those of the pristine PP separator. The excellent mechanical performance of LLZO@PP is mainly attributed to the continuous and interconnected LLZO nanofiber network. The rigid ceramic framework effectively homogenizes external stress distribution and restrains crack initiation and propagation, thereby improving the overall tensile performance of the composite separator, even with minor interfacial microcracks between the LLZO coating and PP substrate. Moreover, the tightly adhered LLZO coating constructs abundant physical anchoring sites on the PP substrate, further strengthening interfacial interaction and enhancing the overall mechanical toughness of LLZO@PP.

Thermal stability is another critical indicator for evaluating the safety performance of LIBs. Thermal shrinkage, structural distortion, melting or structural breakdown of separators under high-temperature conditions may induce direct contact between the anode and cathode, thereby triggering severe thermal runaway and even battery failure. Figure 5b shows photos of PP, C-LLZO@PP and LLZO@PP separators after thermal treatment at different temperatures for 30 min. The pristine PP separator undergoes substantial thermal shrinkage at 140 °C, with deformation further aggravated at 160 °C, which originates from the inherent poor thermal resistance of PP substrates. By contrast, both C-LLZO@PP and LLZO@PP exhibit suppressed dimensional shrinkage at 140 °C and 160 °C, among which LLZO@PP retains the most favorable dimensional integrity. This improvement is attributed to the rigid framework constructed by the high-thermal-stability of the LLZO nanofiber layer coated on the separator surface, which restricts the violent movement of PP molecular chains at high temperatures. Although the two LLZO-modified separators still produce observable thermal contraction under thermal treatment, the intrinsically excellent thermal robustness of garnet-type LLZO effectively mitigates excessive shrinkage and structural distortion, thereby maintaining the basic structural integrity of composite separators.

The thermal properties of different separators were analyzed via thermogravimetric (TG) analysis. As shown in Figure 5c, the thermal decomposition profile of LLZO@PP can be divided into four distinct temperature regions: Region I (below 100 °C), Region II (100–450 °C), Region III (450–500 °C) and Region IV (500–700 °C). The pristine PP separator starts to display a weight loss near 450 °C, originating from the breakage and thermal decomposition of PP molecular chains. In the range of 450–500 °C, the PP separator shows a steep weight loss, which is due to the backbone cleavage of PP molecular chains at higher temperatures [27]. LLZO@PP also exhibits a slight initial weight loss in Region I, corresponding to the desorption of residual moisture adsorbed within the LLZO nanofibers. The weight loss observed in Region II mainly stems from the dehydroxylation of the LLZO crystal structure. The pronounced weight reduction in Region III corresponds to the intrinsic decomposition of the PP matrix, driven by the breakage of C-C covalent bonds. The secondary weight loss in Region IV is ascribed to the high-temperature phase transition of garnet-type LLZO. Notably, the PP separator exhibits abrupt mass loss close to 490 °C and leaves nearly no residual char upon heating to high temperatures. In contrast, LLZO@PP retains a residual weight of 33.46% at 800 °C, further confirming its improved thermal stability.

The electrolyte uptake of separators is closely related to their surface chemical polarity and interfacial affinity toward the liquid electrolyte, and this parameter can be calculated via Equation (S3). Separators with high electrolyte uptake can stabilize the electrochemical environment during long-term cycling, enabling LIBs to realize efficient and durable operation. As shown in Figure 5d, the PP separator delivers the lowest electrolyte uptake of 109.1 ± 5%. Benefiting from the inherent lithiophilic characteristics of garnet-type LLZO nanofibers, LLZO@PP achieves the highest electrolyte uptake of 193.45 ± 6.7%, exceeding that of C-LLZO@PP (168.23 ± 7.2%). This enhancement stems from abundant polar and lithiophilic active sites on LLZO surfaces, which improve the electrolyte wettability and interfacial affinity of the modified separator. The favorable electrolyte affinity of LLZO@PP reduces electrode-separator interfacial impedance and boosts overall ionic conductivity, benefiting its comprehensive electrochemical performance.

Electrolyte wettability represents another vital factor governing the electrochemical performance of separators, which is generally evaluated via electrolyte contact angle measurements. Satisfactory electrolyte wettability enables rapid electrolyte infiltration and uniform liquid distribution on the separator surface, which accelerates Li^+^ migration and mitigates interfacial polarization. As shown in Figure 5e, the PP separator presents inferior electrolyte wettability with an initial contact angle of 39.9°, which only slightly decreases to 36° after 1 s. In comparison, both LLZO-modified separators exhibit distinctly improved wetting performance. Both C-LLZO@PP and LLZO@PP achieve full electrolyte spreading, with the contact angle falling to 0° within 1 s. Notably, LLZO@PP delivers the smallest initial contact angle of 20° and the fastest wetting kinetics. Such favorable wettability originates from the intrinsic lithiophilic characteristic and abundant polar sites of garnet LLZO nanofibers, which effectively improve interfacial electrolyte affinity. Moreover, the interconnected network of the LLZO nanofiber coating optimizes electrolyte infiltration and liquid retention behavior, further facilitating rapid ion transport and improving the long-term cycling stability of batteries.

### 3.5. Electrochemical Performances

**Figure 6:**
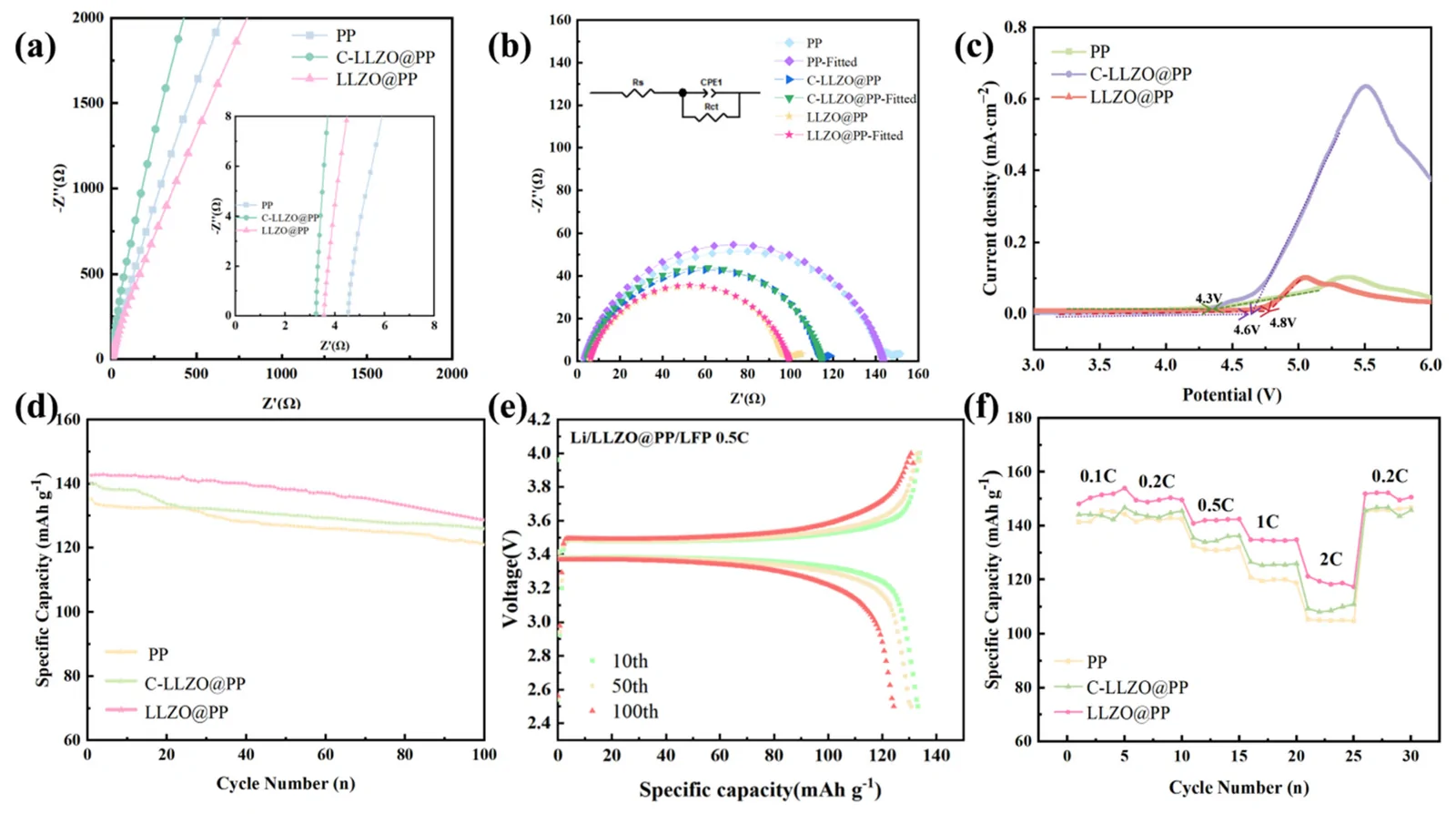
(**a**) EIS Nyquist plots of SS|separator|SS symmetric cells. (**b**) Interfacial impedance extracted from Li|separator|Li symmetric cells. (**c**) LSV curves of SS|separator|Li cells measured at a scan rate of 1 mV s^−1^. (**d**) Cycling performance at 0.5 C, (**e**) GCD profiles at selected cycles, and (**f**) rate performance of LiFePO_4_|separator|Li half-cells.

High ionic conductivity facilitates rapid Li^+^ transport at high current densities and effectively improves the rate capability of LIBs. Figure 6a demonstrates electrochemical impedance spectroscopy (EIS) Nyquist plots of stainless-steel symmetric cells (SS|separator|SS) assembled with different separators. The bulk resistance ($R_{b}$) of each separator is directly determined from the high-frequency intercept on the real axis of the Nyquist plots. According to Equation (S4), the ionic conductivity (δ) of the separators was calculated from the bulk resistance and separator thickness, and the corresponding values are summarized in Table 1. Both LLZO-modified separators exhibit higher ionic conductivity than the pristine PP separator (0.215 mS cm^−1^), which is attributed to their porous network structure, improved electrolyte uptake, and favorable electrolyte wettability. Specifically, C-LLZO@PP displays an ionic conductivity of 0.481 mS cm^−1^, whereas LLZO@PP delivers the highest ionic conductivity of 0.571 mS cm^−1^. The improved conductivity of LLZO@PP originates from the intrinsic lithiophilic nature and Li^+^-conducting characteristics of LLZO nanofibers, which promote full electrolyte infiltration throughout the separator matrix, lower the Li^+^ migration energy barrier, and consequently raise the overall ionic conductivity of the composite separator.

Electrochemical impedance spectroscopy (EIS) was employed to assess the electrode–electrolyte interfacial compatibility of various separators. Figure 6b displays the interfacial resistance of Li metal symmetrical cells (Li|separator|Li) assembled with different separators, which are 140.6 Ω (PP), 110.1 Ω (C-LLZO@PP), and 93.74 Ω (LLZO@PP), respectively. The results confirm that LLZO@PP exhibits optimal interfacial compatibility with both the electrolyte and Li metal electrode, enabling efficient Li^+^ migration and improving the overall electrochemical performance of batteries. This improvement is primarily attributed to the favorable lithiophilicity of the LLZO nanofiber coating toward the liquid electrolyte, which may potentially homogenize Li^+^ flux distribution and mitigate undesirable interfacial side reactions. Additionally, the decreased interfacial impedance of LLZO-modified separators also benefits from their relatively uniform pore structure and favorable electrolyte retention capability. The lithium-ion transference number ($t_{{\mathrm{Li}}^{+}}$) serves as a critical parameter to evaluate the lithium-ion transport capability of separators, which is determined by combining chronoamperometry and EIS tests. As shown in Figure S2c,d, the $t_{{\mathrm{Li}}^{+}}$ values of PP and LLZO@PP separators are calculated according to Equation (S5), yielding values of 0.14 and 0.46, respectively. This significant enhancement originates from the intrinsic Li^+^-conducting characteristic of LLZO and the continuous interconnected nanofiber network. The incorporation of LLZO nanofibers effectively increases $t_{{\mathrm{Li}}^{+}}$, facilitating faster and more uniform Li^+^ transport within the composite separator.

A broad electrochemical stability window is essential for separators to suppress irreversible oxidative degradation and maintain stable internal chemical environments during long-term cycling. Linear sweep voltammetry (LSV) tests were performed on SS|separator|Li cells to evaluate the oxidation-onset potential of different separators. As shown in Figure 6c, the oxidation-onset potentials are approximately 4.3 V (PP), 4.6 V (C-LLZO@PP), and 4.8 V (LLZO@PP), respectively. Notably, C-LLZO@PP delivers a distinctly elevated anodic current after oxidation onset, reaching around 0.7 mA, which points to undesirable oxidative side reactions. Although C-LLZO@PP shows a moderately higher oxidation-onset potential than PP, its increased oxidation current reveals inadequate anodic stability. In comparison, LLZO@PP maintains a relatively low anodic current after the oxidation-onset point, reflecting a weaker anodic response compared with C-LLZO@PP. This suggests that LLZO-nanofiber modification increases the oxidation-onset potential of the commercial PP separator, indicating improved high-voltage tolerance toward the liquid electrolyte.

Cycling and rate performances of LIBs are key indicators for evaluating the practical application potential of separators. Figure 6d shows the cycling performance of LiFePO_4_|separator|Li half-cells employing different separators at 0.5 C for 100 cycles. The half-cell assembled with LLZO@PP delivers a high initial discharge specific capacity of 143 mAh g^−1^ and achieves optimal capacity retention of 90.21% after 100 cycles. The half-cell with C-LLZO@PP exhibits a moderate initial specific capacity of 140 mAh g^−1^ and a capacity retention of 90.17%, whereas the half-cell using pristine PP displays the lowest initial discharge capacity (135 mAh g^−1^) and the lowest capacity retention of only 88.6%. Notably, the LLZO@PP-assembled half-cell exhibits a much slower capacity decay during cycling relative to the other two groups. This improvement may arise from the lithiophilic nature and fast Li^+^ transport pathways of the LLZO nanofiber coating, which may homogenize Li^+^ flux, inhibit lithium dendrite growth, alleviate persistent parasitic reactions at the electrode–electrolyte interface, and stabilize the half-cell structure during repeated lithiation and delithiation.

Galvanostatic charge–discharge (GCD) profiles of the LLZO@PP-assembled half-cell at the 10th, 50th and 100th cycles (0.5 C) are displayed in Figure 6e, intuitively revealing the evolution of discharge capacity and electrochemical polarization upon long-term cycling. As cycling proceeds, a slight attenuation of discharge capacity and a subtle widening of the charge–discharge voltage gap are observed, indicating a progressive polarization accumulation. Even after 100 cycles, the LiFePO_4_|LLZO@PP|Li half-cell still maintains flat and stable charge/discharge voltage platforms, demonstrating suppressed interfacial degradation and well-preserved redox reaction kinetics [28]. The long-term cycling performance of the LiFePO_4_|LLZO@PP|Li half-cell is further evaluated at 1 C over 100 cycles, with the corresponding capacity retention and Coulombic efficiency profiles presented in Figure S2a. The half-cell delivers an initial discharge specific capacity of 135 mAh g^−1^, a capacity retention of 93.3%, and a Coulombic efficiency maintained near 100% throughout the cycling test. This result further verifies the favorable cycling durability of the LLZO nanofiber-modified separator under a relatively high-rate operation. Post-mortem SEM images of PP, C-LLZO@PP and LLZO@PP separators after 100 cycles are shown in Figure S3. Impressively, the cycled LLZO@PP separator maintains a relatively intact surface with uniform lithium deposition and no obvious lithium-dendrite formation.

The rate capabilities of LiFePO_4_|separator|Li half-cells with different separators are further compared in Figure 6f. The discharge specific capacity of all assembled half-cells gradually decreased with increasing current rate. However, at identical current densities, the LiFePO_4_|LLZO@PP|Li half-cell always delivers the highest discharge capacity. Even at a high rate of 2 C, it still retains a substantial discharge specific capacity of 119 mAh g^−1^. Such performance originates from the lowered interfacial impedance and enhanced electrolyte affinity conferred by the LLZO nanofiber coating. Considering that the initial 0.1 C cycle merely acts as an electrode activation step rather than a valid benchmark for capacity evaluation, the baseline capacity is defined using the subsequent 0.2 C stage. Notably, when the current density reverts from 2 C to 0.2 C, the recovered discharge capacity is marginally higher than that measured during the initial 0.2 C segment. This observation might be ascribed to sufficient electrolyte infiltration and progressive electrode activation during cycling. This indicates that the LLZO nanofiber-modified separator preserves favorable electrochemical performance after repeated charge-discharge cycles under variable current densities. Furthermore, the Coulombic efficiency of the LiFePO_4_|LLZO@PP|Li half-cell stays close to 100% throughout the rate test (Figure S2b). Overall, the improved cycling stability and rate capability of the LLZO@PP separator stem from lowered interfacial resistance, enhanced ionic conductivity, and accelerated Li^+^ transport.

**Figure 7:**
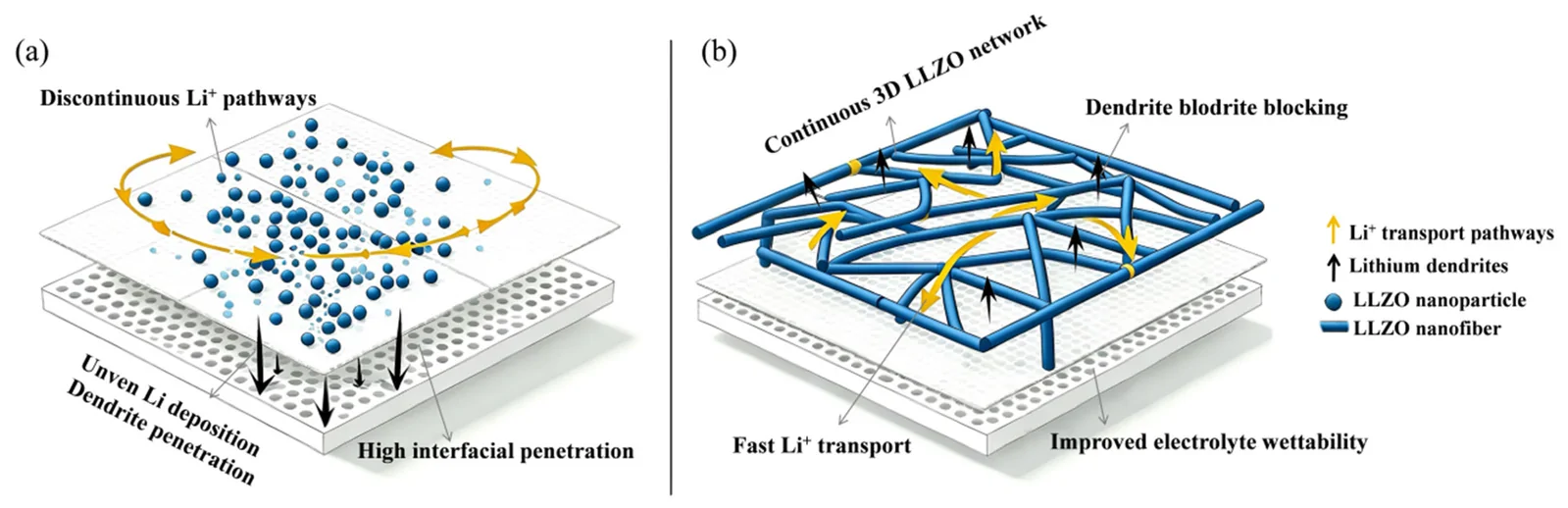
Comparison of working mechanisms for PP separators coated with (**a**) LLZO nanoparticles and (**b**) LLZO nanofibers.

To clarify the structural origins underlying the divergent electrochemical performances, Figure 7 schematically illustrates the working mechanisms of PP separators modified with LLZO nanoparticles and LLZO nanofibers (C-LLZO@PP and LLZO@PP). Discrete C-LLZO nanoparticles are randomly dispersed throughout the coating layer, generating abundant interfacial voids and discontinuous Li^+^ transport pathways. Such a fragmented architecture leads to sluggish ion migration, insufficient electrolyte retention, and uneven lithium deposition, making the separator susceptible to dendrite penetration. In sharp contrast, high-aspect-ratio LLZO nanofibers construct a continuous and interconnected three-dimensional network on the PP surface. This integrated LLZO nanofiber skeleton provides uninterrupted fast Li^+^ transport pathways, improves electrolyte wettability, and lowers interfacial resistance. Furthermore, the rigid crosslinked nanofiber network physically suppresses dendrite propagation and restricts thermal shrinkage of the PP substrate, thereby enhancing the cycling stability and thermal safety of LIBs [29].

## 4. Conclusions

In this work, uniform cubic-phase LLZO nanofibers are successfully fabricated via SSFSE followed by sintering at 780 °C. A PVP concentration of 10 wt% is verified as the optimal spinning parameter for obtaining bead-free and homogeneous precursor nanofibers. The LLZO nanofiber-coated PP separator (LLZO@PP) is prepared via blade coating, while the LLZO nanoparticle-modified separator (C-LLZO@PP) serves as the control group. Unlike discrete C-LLZO nanoparticles that form discontinuous conductive pathways, the interwoven LLZO nanofibers construct a continuous three-dimensional network. This interconnected structure offers long-distance Li^+^ transport pathways, abundant uniform lithiophilic sites and a thermally stable rigid skeleton, endowing LLZO@PP with improved electrolyte affinity, ionic conductivity and thermal dimensional stability compared with its nanoparticle-modified counterpart.

The optimized LLZO@PP exhibits a high electrolyte uptake of 193.45 ± 6.7%, fast full electrolyte spreading within 1 s, and an ionic conductivity of 0.571 mS cm^−1^. LSV measurements confirm that the LLZO@PP separator presents an oxidation-onset potential up to 4.8 V. The Li//LiFePO_4_ half-cell assembled with LLZO@PP delivers an initial discharge capacity of 143 mAh g^−1^ with 90.21% capacity retention after 100 cycles at 0.5 C, and retains a high capacity of 119 mAh g^−1^ at 2 C. This work demonstrates a facile laboratory-scale coating strategy for the modification of commercial PP separators. Compared with LLZO nanoparticle-modified separators, electrospun LLZO nanofiber coatings impart PP separators with comprehensively improved electrochemical and thermal performances under the tested conditions. Nevertheless, further systematic investigations are still required to validate the practical applicability of such nanofiber-modified separators for future battery applications.

## Figures and Tables

**Table 1:** $R_{b}$ and δ of cells assembled with PP, C-LLZO@PP and LLZO@PP separators.

Separators	PP	C-LLZO@PP	LLZO@PP
Thickness (mm)	0.025	0.040	0.052
$R_{b}$ (Ω)	4.541	3.248	3.559
δ (mS/cm)	0.215	0.481	0.571

## Data Availability

The original contributions presented in this study are included in the article/Supplementary Materials. Further inquiries can be directed to the corresponding author.

## Supplementary Materials

The following supporting information can be downloaded at: https://www.mdpi.com/article/10.3390/ma19183991/s1, Materials; preparation of spinning solution; characterization; battery assembly and electrochemical tests; Figure S1: XPS survey spectrum of LLZO nanofibers. Figure S2: (a) Cycling performance at 1 C and (b) rate performance of the LiFePO4|LLZO@PP|Li half-cell. EIS spectra and current variation curves before and after polarization of Li|separator|Li cells assembled with (c) pristine PP and (d) LLZO@PP separators. Figure S3: SEM images of (a) PP, (b) C-LLZO@PP, and (c) LLZO@PP separators before cycling, as well as (d–f) their corresponding post-mortem morphologies after 100 cycles at 0.5 C.
