# Supplementary material for: Li7La3Zr2O12 Nanofiber-Modified Polypropylene Composite Separator for High-Performance Lithium-Ion Batteries
Source: Materials (Basel). 2026 Sep 19;19(18):3991. doi: 10.3390/ma19183991 (PMC13608711; doi:10.3390/ma19183991)
Supplement: Supplementary file 1 [file materials-19-03991-s001.zip › materials-4512653-supplementary.pdf]

$Li_7La_3Zr_2O_{12}$ Nanofiber-Modified Polypropylene Composite Separator for High-performance Lithium-ion Batteries

Yuxing Liu [1], Lan Xu[1,2,3*]

[1]*National Engineering Laboratory for Modern Silk, College of Textile and Engineering, Soochow University, Suzhou, 215123, China*

[2]*Jiangsu Engineering Research Center of Textile Dyeing and Printing for Energy Conservation, Discharge Reduction and Cleaner Production, Soochow University, Suzhou 215123, China*

3Jiangsu Advanced Textile Engineering Technology Center, Jiangsu, Nantong, 226007, China

** Corresponding authors: lanxu@suda.edu.cn (L.X.).*

Materials

Zirconyl nitrate ($ZrO(NO_3)_2$, AR, ≥99%), lanthanum nitrate hexahydrate ($La(NO_3)_3\cdot 6H_2O$, AR, ≥99%), and lithium nitrate ($LiNO_3$, AR, ≥99%) were purchased from Shanghai Macklin Biochemical Co., Ltd., Shanghai Eno Chemical Technology Co., Ltd., and Shanghai Titan Scientific Co., Ltd. (China), respectively. Poly(vinyl pyrrolidone) (PVP, Mw ≈ 1300000), N,N-dimethylformamide (DMF, AR, 98%), and acetic acid (AR, 99%) were obtained from Shanghai Meryer Chemical Technology Co., Ltd., Shanghai Chemical Reagent Co., Ltd., and Jiangsu Qiangsheng Functional Chemistry Co., Ltd. (China), respectively. Poly(vinylidene fluoride) (PVDF, 99.9%) and N-methyl-2-pyrrolidone (NMP, 99.8%) were supplied by Shenzhen Kejing Star Technology Co., Ltd. (China). Commercial Celgard 2500 polypropylene (PP) separator, fabricated via a dry-stretching process with a nominal thickness of 25 μm and an average pore diameter of 0.064 μm, was purchased from Suzhou Sinero Technology Co., Ltd. (China). The liquid electrolyte was a 1 M lithium hexafluorophosphate ($LiPF_6$) solution dissolved in a ternary mixed solvent of ethylene carbonate, dimethyl carbonate, and ethyl methyl carbonate (EC/DMC/EMC, 1:1:1 by volume), which was provided by Suzhou Dodo Chemical Technology Co., Ltd. (China). For electrode preparation, PVDF,

conductive carbon black (Super P-C45), $LiFePO_4$ powder, and NMP were obtained from Ferro Corp.

Preparation of Spinning Solution

DMF and acetic acid were thoroughly mixed at a mass ratio of 5:1 to form the solvent. Afterwards, 2 mmol of $ZrO(NO_3)_2$, 7.7 mmol of $LiNO_3$, and 3 mmol of $La(NO_3)_3 \cdot 6H_2O$ were added to the solvent sequentially under magnetic stirring. A 10% excess of $LiNO_3$ was used to compensate for lithium loss caused by volatilization during calcination. Once all inorganic salts were completely dissolved, PVP was introduced into the solution at weight fractions of 8 wt%, 10 wt% and 12 wt%, respectively. The mixed solution was stirred overnight to achieve complete dissolution of PVP, thus obtaining a homogeneous electrospinning precursor solution for subsequent processing.

Characterization of Separators

The morphology of nanofiber membranes (NFMs) was observed through scanning electron microscopy (SEM, Regulus8100 and Hitachi S4800, Hitachi, Japan). For nanofiber diameter distribution analysis, five SEM images were randomly selected, and twenty nanofibers within each image were measured via ImageJ software. The crystalline structure of samples was elucidated using X-ray diffraction (XRD, D8 Advance, German) analysis with a diffraction angle (2θ) in the range of 10°-70°. The chemical composition and elemental valence state of separators were characterized by X-ray photoelectron spectroscopy (XPS, Thermo Scientific K-Alpha, USA) with Al Kα radiation as the excitation source. The thermal stability of samples was measured by thermogravimetric analysis (TGA) from room temperature to 800 °C at a heating rate of 20 °C min^{-1} under a N_2 atmosphere (TG/DTA, TG/DTA5700, USA). To evaluate the thermal shrinkage behavior of nanofiber separators, separators with a diameter of 19 mm were placed in a constant temperature drying oven and held at various temperatures for 30 min at each testing point.

A universal material testing machine (Instron-3365) was used to test the mechanical properties of the samples. All tensile samples were cut along the intrinsic

stretching direction of the commercial PP membrane to eliminate orientation-induced mechanical discrepancies. The size of the test sample was 40 mm×10 mm. Before the test, the thickness of each separator was measured at multiple positions using a digital thickness gauge, and the average thickness was employed to determine the cross-sectional area. All samples were tested in triplicate, and the average value was collected for statistical analysis. During the test, the clamping gauge distance was fixed at 20 mm with a constant tensile rate of 20 mm/min. The fracture stress and strain of separators were calculated according to the following formulas:

$$\delta=\frac{P}{A} \tag{S1}$$

where δ is the stress (MPa) of the separator, $P$is the load (N), and A is the cross-sectional area (mm^2) of the sample.

$$\varepsilon=\frac{L\text{-}L_0}{L_0}\times100\% \tag{S2}$$

where ε is the strain (%) of the separator, $L_0$is the original length (mm) of the specimen, L is the length (mm) of the specimen after deformation, and $L\text{-}L_0$ is the displacement tested by the universal testing machine.

The electrolyte affinity of separators was evaluated by electrolyte contact angle measurements using a contact angle goniometer (Krüss DSA 100, Krüss GmbH, German), and the volume of each droplet of electrolyte was set to 5 μL. Each sample was measured at five different positions, and the average contact angle was calculated. In addition, the electrolyte uptake (U_{Uptake}) of separators with a diameter of 19 mm was tested by soaking in electrolyte for 2 h, and calculated according to the following formula (S3):

$$U_{Uptake}=\frac{M_{\mathrm{h}}\text{-}M_q}{M_q}\times100\% \tag{S3}$$

where M_h is the weight of the separator after absorbing the electrolyte and M_q is the weight of the dry separator.

Battery Assembly

The $LiFePO_4$ cathode was prepared via a conventional slurry-coating method. First, PVDF and carbon black were sequentially dispersed in NMP solvent and

magnetically stirred for 2 h to form a homogeneous mixture. Subsequently, $LiFePO_4$ active material was incorporated into the above dispersion, followed by continuous stirring for 8–10 h to obtain a uniform cathode slurry. The well-mixed slurry was then cast onto aluminum foil current collectors. After coating, the electrodes were dried at 110 °C for 12 h to completely remove residual solvent and stabilize the electrode structure. Finally, flat and uniform cathode discs were obtained, with the areal loading of active material controlled at approximately 2 mg cm^{-2}.

Battery casings were ultrasonically cleaned twice with ethanol and dried at 60 °C, and then placed in a glove box filled with argon gas (both the O_2 and H_2O content were less than 0.01 ppm). All composite separators and $LiFePO_4$ cathode were dried for 30 min to eliminate residual moisture before battery assembly.

Electrochemical Tests

Ionic conductivity can characterize the ability of separators to allow lithium-ions (Li^+) to pass through. For measurement of the ionic conductivity of separators, a separator was placed between two stainless steel (SS) electrodes and assembled into a coin cell (CR2025) in a glove box filled with argon. Then a electrochemical workstation (SP-300, Bio-Logic company, France) was applied to test the symmetric cell (SS|separator|SS battery) by using the alternating current (AC) impedance method. AC impedance spectra of cells were recorded over a frequency range of 1 to 10^6 Hz with a voltage amplitude of 5 mV. The ionic conductivity of separators (δ) can be calculated using the following equation:

$$\delta=\frac{L}{R_b\times A} \tag{S4}$$

where L is the thickness (cm) of separators, R_b is the bulk resistance (Ω) directly extracted from the high-frequency intercept on the real axis of the Nyquist plot, and A is the effective area of the SS electrode (1.6×1.6 cm^2).

Electrochemical impedance spectroscopy (EIS) was employed to assess the electrode–electrolyte interfacial compatibility of different separators. Li-metal symmetric cells (Li|separator|Li) were assembled into a coin battery (CR2032), and

their interfacial electrochemical behavior was characterized using an electrochemical workstation (SP-300). EIS measurements were conducted via the potentiostatic EIS (PEIS) module with an AC perturbation amplitude of 5 mV and a scanning frequency range of 0.1–10^5 Hz. For measurement of the lithium ion transference number (t_{Li^+}), Li|separator|Li symmetric cells were tested on the SP-300 workstation under a polarization voltage (ΔV) of 10 mV. t_{Li^+} was calculated according to the following equation:

$$t_{Li^+} = \frac{I_S R_b^0[\Delta V - I_0 R_1^0]}{I_0 R_b^s[\Delta V - I_S R_1^s]} \quad \text{(S5)}$$

where I_0 and I_s are the initial and steady-state currents obtained from chronoamperometry, respectively, R_b^0 and R_b^s are the bulk resistances before and after potentiostatic polarization, and R_1^0 and R_1^s are the interfacial resistances before and after potentiostatic polarization.

Linear sweep voltammetry (LSV) was utilized to evaluate the electrochemical stability window of the separators. Based on the coin battery assembled with SS as the working electrode and Li metal as the counter electrode (SS|separator|Li), LSV tests were carried out using the electrochemical workstation (SP-300) at a scanning rate of 1 mV s^{-1} over a voltage range of 0–6 V at room temperature. The working electrode area was 1.6×1.6 cm^2, and 120 μL of electrolyte was applied for each cell. The electrochemical stability window was determined from the onset potential of the abrupt current increase in the LSV curves, which corresponded to the oxidative decomposition of the separator–electrolyte system.

The cycling and rate performances of $LiFePO_4$|separator|Li half-cells were tested on a battery testing system (CT-4008T-5V10Ma-164, Shenzhen Xinwei Electronics Co., Ltd, China). Galvanostatic cycling was performed within a potential window of 2.5-4.0 V at 0.5 C. In order to evaluate the rate capability, the $LiFePO_4$|separator|Li cells were galvanostatically cycled at various current densities ranging from 0.1 C, 0.2 C, 0.5 C to 2 C. All cells were charged to 4.0 V and discharged to 2.5 V at room temperature. The C-rate is defined as the current required to fully discharge a fully charged cell within 1/n hours.

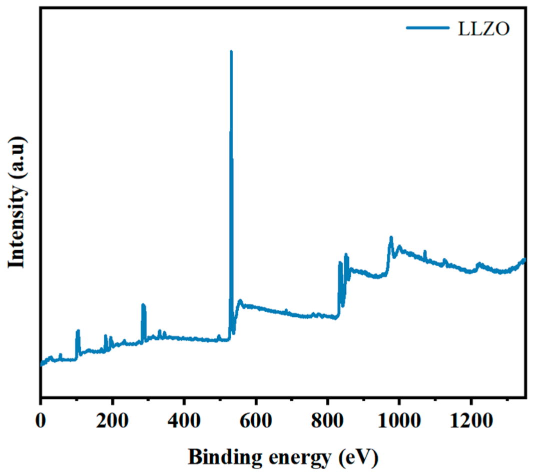

Figure S1. XPS survey spectrum of LLZO nanofibers.

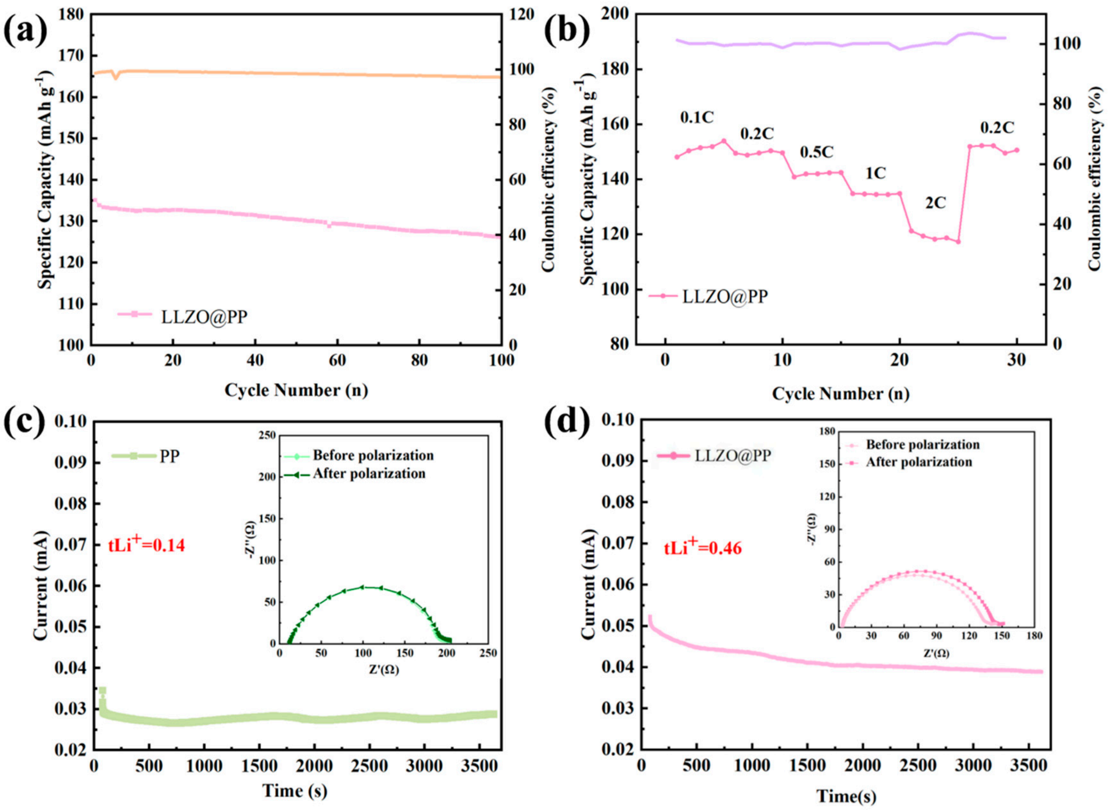

Figure S2. (a) Cycling performance at 1 C and (b) rate performance of the $LiFePO_4$|LLZO@PP|Li half-cell. EIS spectra and current variation curves before and after polarization of Li|separator|Li cells assembled with (c) pristine PP and (d) LLZO@PP separators.

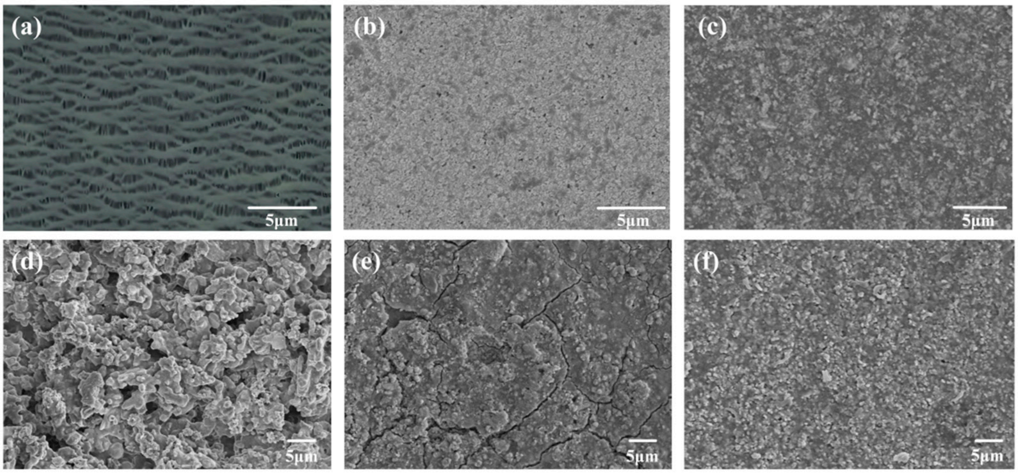

Figure S3. SEM images of (a) PP, (b) C-LLZO@PP, and (c) LLZO@PP separators before cycling, as well as (d-f) their corresponding post-mortem morphologies after 100 cycles at 0.5 C.
